# Advances in Research on the Genotype–Phenotype Correspondence of Antibiotic Resistance in *Helicobacter pylori* and Its Molecular Mechanisms: A Narrative Review

**DOI:** 10.1155/grp/4557969

**Published:** 2026-10-26

**Authors:** Hui Gong, Yan Chun Huang, Bo Yang, Zhen Yu Song, Xue Qiong Xu, Fang Cui Xiao, Rong Peng, Chang Ping Li

**Affiliations:** ^1^ Department of Gastroenterology West China Longquan Hospital Sichuan University/The First People′s Hospital of Longquanyi District Chengdu Chengdu Sichuan China; ^2^ Department of Laboratory Medicine West China Longquan Hospital Sichuan University/The First People′s Hospital of Longquanyi District Chengdu Chengdu Sichuan China; ^3^ Department of Endoscopy Center West China Longquan Hospital Sichuan University/The First People′s Hospital of Longquanyi District Chengdu Chengdu Sichuan China; ^4^ Department of Gastroenterology Affiliated Hospital of Southwest Medical University Luzhou Sichuan China ahswmu.cn

**Keywords:** antibiotic resistance, *Helicobacter pylori*, individualized treatment, molecular mechanism, resistance genotype, resistance phenotype

## Abstract

*Helicobacter pylori* (*H. pylori*), recognized as the primary pathogen responsible for chronic gastric infections, has exhibited an increasing prevalence of antibiotic resistance, which significantly impairs the efficacy of clinical treatments. In recent years, the extensive utilization of antibiotics alongside the rising prevalence of resistant strains has elevated the investigation of the resistance mechanisms of *H. pylori* to a prominent area of research. This article systematically examines the phenotypic characteristics associated with the antibiotic resistance of *H. pylori*, establishing a correspondence with resistance genotypes. Particular emphasis is placed on analyzing the resistance mechanisms linked to commonly prescribed antibiotics such as clarithromycin, metronidazole, and amoxicillin, in addition to the molecular characteristics of the relevant resistance genes (such as mutations in “gyrA,” “gyrB,” “23S rRNA,” “frxA,” “pbp1,” and “rdxA”). By merging advancements in molecular biology with findings from epidemiological studies, this review explores the potential application of resistance genotype detection in facilitating tailored clinical treatment approaches. The objective of this review is to enhance the understanding of the resistance mechanisms of *H. pylori*, fostering the development of precise diagnostic and therapeutic strategies and offering theoretical insights and future research pathways for the treatment of anti–*H. pylori* infections.

## 1. Introduction

*Helicobacter pylori* (*H. pylori*), a globally prevalent gram‐negative spiral bacterium, exhibits distinct epidemiological disparities tied to geography, ethnicity, and age. A 5‐year retrospective study in Karachi, Pakistan, found declining infection rates linked to gastric mucosal pathology [1], whereas research in ethnically diverse Lod, Israel, pointed to genetic and nonenvironmental influences on infection variability [2]. In eastern Morocco, childhood infection rates rose sharply between ages 10–16, often coinciding with active gastric inflammation, emphasizing the need for early intervention [3]. Overall, *H. pylori* is a major risk factor for gastrointestinal disorders and gastric cancer, shaped by environmental and genetic factors [4, 5].

In clinical treatment, antibiotic therapy is the core method for *H. pylori* eradication. However, the emergence of antibiotic resistance has greatly restricted the improvement of eradication rates, becoming one of the biggest challenges in current treatment [6]. Studies have shown that the resistance rates of *H. pylori* to commonly used antibiotics such as clarithromycin, metronidazole, and levofloxacin are increasing. A Spanish childhood study recorded 44.9% clarithromycin resistance, including dual resistance cases [7], whereas ampicillin resistance, though lower, requires ongoing monitoring because of widespread use [8]. To address this, guidelines recommend bismuth‐containing quadruple therapy or novel agents like vonoprazan, alongside individualized strategies based on drug sensitivity testing or local epidemiological data [9–11].

Resistance mechanisms primarily involve gene mutations targeting antibiotic action: 23S rRNA mutations (A2146G/A2147G) drive clarithromycin resistance, whereas DNA gyrA mutations cause quinolone resistance [8, 12, 13]. vacA gene allelic variations also correlate with bacterial virulence [14]. Genotype–phenotype correlations support genetic testing as a resistance predictor, though discrepancies exist because of gene silencing, epigenetic regulation, or polygenic effects [8, 15, 16]. Combining genetic testing with traditional drug sensitivity assays enhances clinical guidance accuracy [9, 17].

Studying resistance phenotype–genotype relationships improves understanding of resistance mechanisms and informs personalized treatment, optimizing antibiotic selection to boost eradication rates and reduce resistant strain transmission [15, 18]. Despite advances in genetic testing, challenges remain in genotype–phenotype matching, standardized detection, and widespread implementation of genotype‐guided therapy [6, 9]. Regional epidemiological data and patient‐specific factors (age and treatment history) must also be integrated into clinical decisions [8, 15].

This review synthesizes progress in *H. pylori* antibiotic resistance research, focusing on gene mutations and phenotypic correlations, their clinical diagnostic and therapeutic value, and existing limitations. It further outlines future research directions and technological trends, aimed at deepening the understanding of resistance mechanisms and support evidence‐based eradication strategies to improve patient outcomes.

## 2. Materials and methods

This is a narrative review.

### 2.1. Literature Selection Approach

A professional search was conducted in English databases (PubMed and BioMed Central) and Chinese databases (China National Knowledge Infrastructure and China Wanfang Database), limiting the time from January 1, 2015, to September 21, 2025. The keywords were as follows: (“Helicobacter pylori” or “Helicobacter Infections” or “h pylori” or “H. pylori” or “helicobacter pylori”) and (“antibiotic resistance” or “Drug Resistance” or “resistant” or “resistance” or “eradication failure” or “antibacterial drug resistance” or “antimicrobial drug resistance”). These search terms were combined with “resistance phenotype” or “resistance genotype” and each individual antibiotic serially (“clarithromycin,” “metronidazole,” “levofloxacin,” “tetracycline,” “quinolone,” and “amoxicillin”). The language of the literature was limited to Chinese or English. The flowchart of study selection is detailed in Figure 1.

**Figure 1:**
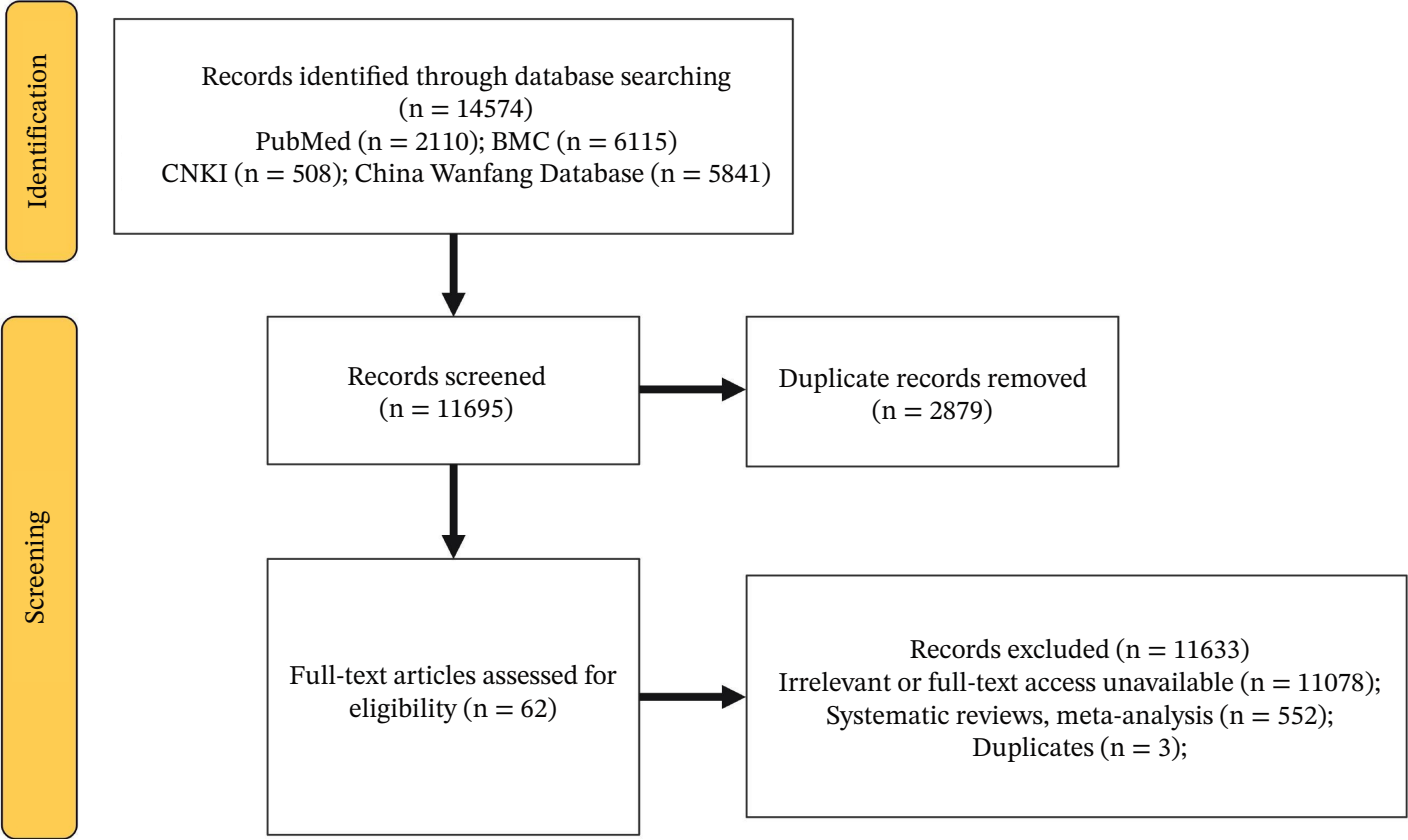
Flowchart of study selection.

### 2.2. Selection Criteria

Inclusion criteria were as follows: (1) the diagnosis of *H. pylori* infection based on at least one of the routine diagnostic methods (the urea breath test, rapid urease test, histology examination, or in vitro culture); (2) no receipt of any antibiotics, proton pump inhibitors, or bismuth within the previous 4 weeks.; (3) antibiotic resistance testing based on at least one of the standard *H. pylori* drug sensitivity tests: the E‐test, the agar dilution method (ADM), the Kirby–Bauer (K–B) disk diffusion test, or polymerase chain reaction (PCR); (4) tests for the primary antibiotics: amoxicillin, levofloxacin, clarithromycin, metronidazole, tetracycline, and furazolidone; and (5) the type of study was a clinical study without limitation.

Exclusion criteria were as follows: (1) studies focusing on special populations, such as diabetic patients and leukemia patients; (2) repetitively published or duplicated data; (3) incomplete basic information and incorrect statistical data; (4) not available for full‐text access; and (5) systematic reviews, meta‐analyses, guidelines, case reports, conference abstracts, experience summaries, author′s replies, scientific and technological achievements, letters to the editor, and trial registry records.

## 3. Phenotypic Characteristics and Detection Methods of *H. pylori* Antibiotic Resistance

### 3.1. Phenotypic Manifestations of Common Antibiotic Resistance

The emergence of clarithromycin resistance presents a significant challenge in the context of antibiotic resistance associated with *H. pylori*, which profoundly influences the efficacy of eradication therapies. Empirical research indicates that the prevalence of clarithromycin resistance exhibits regional variability, yet there is a discernible upward trend overall. For instance, the resistance rate in Egypt is estimated to be around 40%, whereas the Daegu‐Gyeongbuk region in South Korea reports a resistance rate surpassing 30% [19, 20]. This resistance to clarithromycin is correlated with increased rates of treatment failure. Resistant strains demonstrate markedly higher failure rates in comparison to their susceptible counterparts, thereby diminishing overall eradication success rates [7]. Furthermore, mutations in resistance genes, such as A2147G located within the 23S rRNA gene, are closely linked to the manifestation of high‐level clarithromycin resistance [21].

The resistance to metronidazole exhibits considerable variation, with its prevalence differing across geographical regions. Globally, the rates of metronidazole resistance are notably elevated, reaching an alarming 100% in Egypt, around 45% in Moscow, Russia, and exceeding 85% in the northwestern part of China [19, 22, 23]. The underlying mechanisms contributing to metronidazole resistance are intricate, encompassing a variety of factors, including specific gene mutations and the presence of scavenging systems for reactive oxygen species [24]. This heterogeneity in metronidazole resistance poses significant challenges to its clinical utilization, whereas the occurrence of dual resistance, particularly with clarithromycin in conjunction with metronidazole, adds further complexity to treatment strategies [22].

The rates of resistance to amoxicillin and other antibiotics are generally low, yet they exhibit significant regional disparities. In the majority of areas, amoxicillin resistance rates fluctuate between 2% and 6%. However, research conducted in Egypt revealed an alarming resistance rate reaching up to 95% [19, 20]. Additionally, resistance to alternative antibiotics, such as levofloxacin and tetracycline, has escalated to varying extents, thereby influencing therapeutic options [25, 26]. The development of amoxicillin resistance predominantly occurs through mutations in the genes encoding target proteins, with minimal correlation to the production of *β*‐lactamase enzymes [24].

In summary, *H. pylori* resistance rates to clarithromycin, metronidazole, and some other antibiotics continue to rise, with diverse resistance phenotypes showing regional characteristics, significantly affecting the efficacy and clinical prognosis of eradication therapy [27, 28].

### 3.2. Antibiotic Susceptibility Testing Techniques

Traditional *H. pylori* culture and drug sensitivity testing are the gold standards for antibiotic susceptibility testing. Culture methods isolate bacteria from gastric mucosal biopsy specimens followed by drug sensitivity testing (such as the E‐test and ADMs), which can directly obtain minimum inhibitory concentration (MIC) data to guide clinical medication [7, 20]. This method is accurate and reliable but has limitations such as difficulty in cultivation, a long cycle (usually several days), and technical complexity, with high dependence on the cultivation of some strains [28]. The diagnostic and clinical workflow for resistance detection in *H. pylori* is detailed in Figure 2.

**Figure 2:**
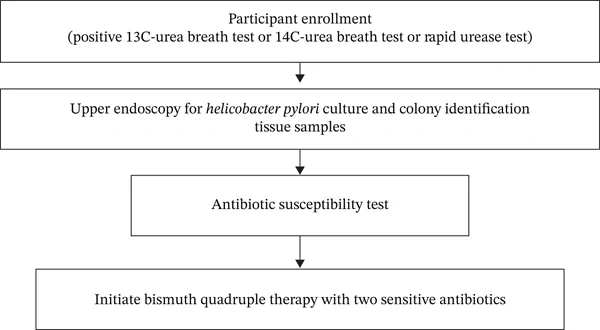
Diagnostic and clinical workflow for resistance detection in *Helicobacter pylori*.

Molecular detection techniques such as PCR and gene chips detect resistance‐related genes to rapidly predict resistance phenotypes. Using PCR to detect 23S rRNA gene mutations for predicting clarithromycin resistance and rdxA and other genes for predicting metronidazole resistance can obtain results within hours [21, 28, 29]. Molecular methods have advantages of rapidity and sensitivity without requiring viable bacterial culture, but their accuracy depends on the coverage of known resistance genes and may miss unknown or complex resistance mechanisms [30].

Emerging high‐throughput sequencing (whole genome sequencing, WGS) technology provides more comprehensive genomic information for resistance phenotype analysis. WGS can simultaneously detect multiple resistance genes and mutations, revealing the diversity and complexity of resistance mechanisms. In addition, WGS can assist in epidemiological tracking to analyze strain transmission and evolution [31]. However, WGS has high costs and data analysis requirements and is currently mainly used in research and large‐scale monitoring projects.

Recent studies have also seen rapid platforms integrating phenotypic and genotypic detection, such as microfluidic chips combining microbial growth detection and gene amplification to complete resistant bacteria detection within 3 h, with both speed and accuracy [32]. In addition, new technologies such as nanomechanical sensors and optical single‐cell detection also show potential in rapid antibiotic susceptibility testing [33, 34].

Overall, current *H. pylori* antibiotic susceptibility testing technologies are gradually developing toward rapidity, precision, and multidimensionality. Traditional culture methods remain the cornerstone of clinical application; molecular detection and high‐throughput sequencing provide powerful tools for resistance prediction and mechanism research, whereas emerging technologies are expected to shorten detection time and enhance clinical guidance value [28, 30].

### 3.3. Clinical Significance of Phenotypic Resistance

The phenotypic resistance exhibited by *H. pylori* plays a pivotal role in influencing the rates of treatment failure. Specifically, resistance to clarithromycin and metronidazole has been linked to a marked decline in eradication success, whereas strains that exhibit MDR are more prone to result in treatment failures [7, 25]. A multicenter study conducted across Europe revealed that patients harboring resistant strains experienced significantly improved eradication rates when treated with regimens tailored to their resistance profiles, as opposed to those receiving empirical treatments [25]. Consequently, the precise identification of phenotypic resistance is essential for the formulation of personalized treatment approaches.

The application of phenotypic resistance testing results in clinical decision‐making is increasingly widespread. According to the Maastricht VI consensus, antibiotic susceptibility testing before treatment is recommended, especially for clarithromycin susceptibility, to guide the selection of first‐line eradication regimens [35]. When testing is unavailable, empirical treatment based on regional resistance rates and variabilities among populations remains a practical choice [7].

However, the long cycle of phenotypic testing limits its rapid clinical application, leading some patients to still receive nontargeted empirical treatment, increasing resistance risks [30]. Although molecular detection applications can shorten time, they cannot completely replace phenotypic testing. Future combination of rapid phenotypic and genotypic detection to improve detection efficiency and accuracy will better serve clinical practice [30, 32].

In addition, phenotypic resistance is also associated with patient clinical characteristics such as previous antibiotic use history, age, and gender, which can assist in clinical prediction of resistance risks [36]. Therefore, comprehensive utilization of phenotypic testing results and clinical information helps optimize treatment regimens, improve *H. pylori* eradication success rates, and reduce resistance incidence [7].

In summary, phenotypic resistance of *H. pylori* significantly affects treatment failure rates, and timely accurate phenotypic resistance detection is key to developing individualized treatment regimens. Currently in clinical practice, phenotypic testing results have gradually been incorporated into treatment decisions, but detection cycles and technical limitations still need to be overcome. Future combination of molecular detection and high‐throughput technologies to achieve rapid and accurate resistance detection will greatly improve clinical treatment outcomes [25, 28].

## 4. *H. pylori* Resistance Genotypes and Their Molecular Mechanisms

### 4.1. Clarithromycin Resistance‐Related Genes and Mutations

Clarithromycin serves as a crucial element in the primary therapeutic regimen aimed at eradicating *H. pylori*; however, there has been a notable global rise in the resistance rates, significantly compromising treatment effectiveness. The development of clarithromycin resistance is predominantly attributed to point mutations within the 23S rRNA gene, with particular emphasis on mutations occurring at the A2146G (previously called A2142G) and A2147G (previously called A2143G) positions, which have been extensively documented as the principal mechanisms of resistance [12, 15, 37]. These specific mutations modify the binding site of clarithromycin on the 23S rRNA, subsequently diminishing the drug′s binding affinity and leading to the manifestation of resistance phenotypes.

Numerous extensive studies have indicated that the A2147G mutation within the 23S rRNA gene exhibits the highest prevalence among clinically resistant strains, demonstrating a strong correlation with clarithromycin resistance [8, 38]. Furthermore, mutations such as A2146G have been validated to exert significant effects on resistance [21, 39]. Additional research has identified other mutations in the 23S rRNA gene, including G2220T and C2248T, which may play a role in resistance; however, their precise roles necessitate further investigation [40].

Beyond mutations in the 23S rRNA gene, specific gene regulatory mechanisms may play a role in clarithromycin resistance. Notably, the hp1181 and hp1184 genes have been identified as linked to resistance in strains from southern China, suggesting their potential utility as early molecular indicators of resistance [41]. Furthermore, bacteria might influence resistance indirectly by modulating the expression of virulence factors, including the cagA and vacA genes [42]. Additionally, it has been observed that resistant strains may undergo compensatory mutations following exposure to clarithromycin, which can enhance their virulence and facilitate biofilm formation, ultimately leading to increased resistance [43].

Research investigating the relationship between genotype and phenotype has demonstrated a strong correlation between mutations A2146G and A2147G in the 23S rRNA gene and the phenotypes of resistance to clarithromycin, which can be utilized as molecular diagnostic targets [12, 38]. Furthermore, the utilization of next‐generation sequencing (NGS) technology has significantly enhanced the precise identification of resistance genotypes, thereby establishing a foundation for personalized therapeutic approaches [44]. Moreover, genetic testing methodologies conducted on gastric juice have been validated to exhibit high sensitivity and specificity in the detection of clarithromycin resistance [45]. The genetic changes of antibiotic resistance in *H. pylori* are detailed in Figure 3 [46].

**Figure 3:**
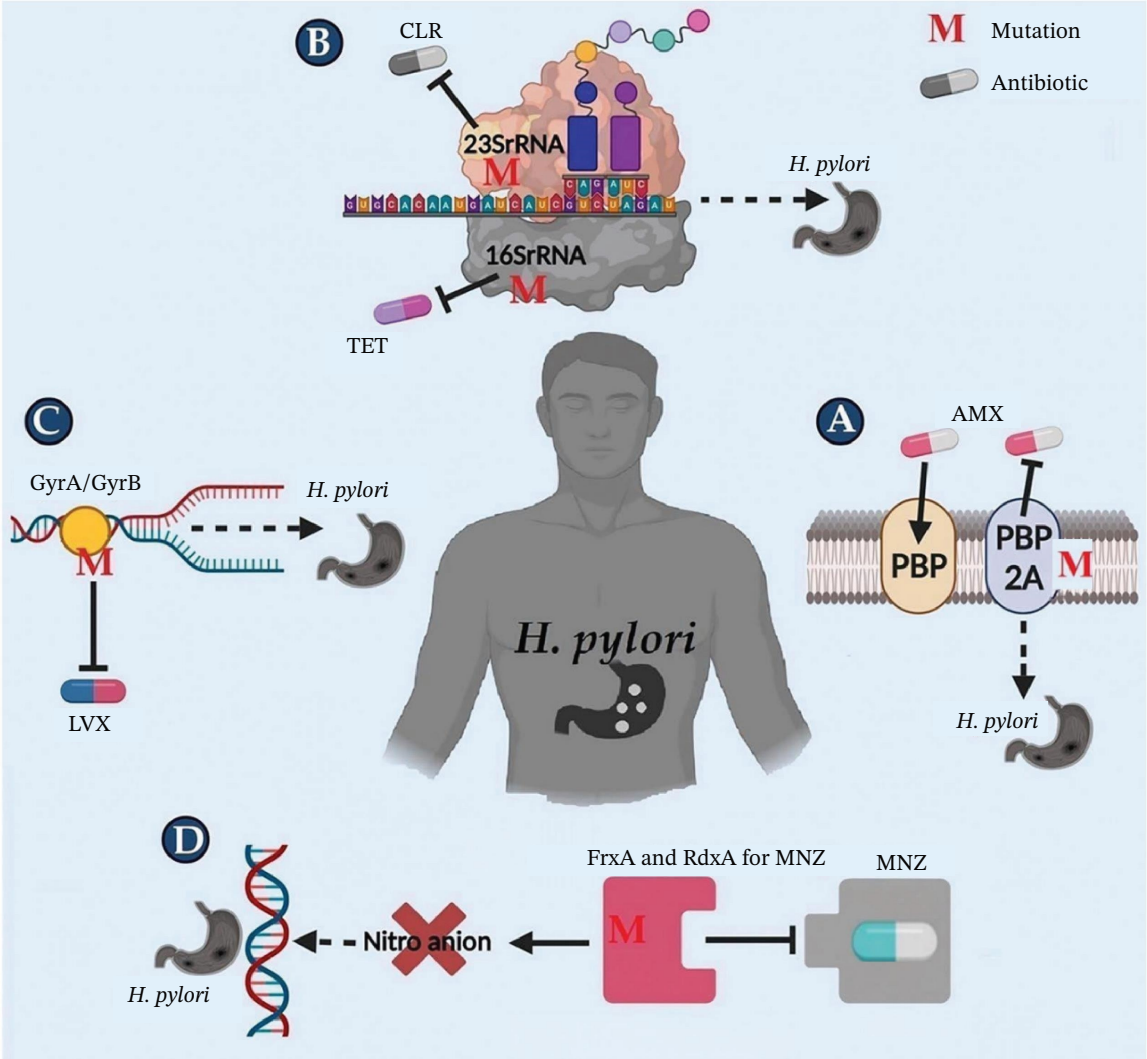
Summary of genes mutations involved in *H. pylori* antibiotic resistance. (A) The mutation of the pbp1 gene is a prevalent method by which *H. pylori* strains obtain resistance to amoxicillin (AMX). (B) The presence of point mutations in the 23S rRNA gene is responsible for clarithromycin (CLR) resistance. The point mutations in the 16S rRNA gene are indicative of tetracycline (TET) resistance. (C) Resistance to levofloxacin (LVX) may be caused by mutations in the genes gyrA and gyrB. (D) Resistance mechanisms of *H. pylori* toward metronidazole (MNZ) are predominantly attributed to single nucleotide mutations in the target genes of frxA and rdxA. This figure was derived from Figure 4 in Reference 46 with minor modifications using BioRender.

In conclusion, the molecular basis underlying clarithromycin resistance in *H. pylori* is primarily attributed to point mutations, specifically A2146G and A2147G, located within the 23S rRNA gene. Additionally, other regulatory mechanisms—including the expression of virulence‐associated genes and efflux pump genes—contribute to this resistance phenomenon [40, 42, 43, 47]. Genotyping has been validated as a reliable approach for predicting resistance, thus offering both theoretical insights and practical assistance for the clinical formulation of individualized treatment strategies.

### 4.2. Metronidazole Resistance Genes and Metabolic Pathway Changes

Metronidazole is an important antibiotic for treating *H. pylori* infection, but its resistance rate remains high, especially prominent in Asian regions [8, 15, 48]. The main molecular mechanism of metronidazole resistance involves reduced activity of metronidazole reductase, mainly caused by mutations in the rdxA and frxA genes [15, 49]. The rdxA gene encodes oxidoreductase involved in metronidazole activation; mutations result in loss of enzyme function, preventing drug reduction activation and producing resistance [50]. Mutations in the frxA gene are also considered auxiliary factors for resistance, especially when rdxA mutations do not fully explain resistance [49].

Multiple studies have found that point mutations, frameshift mutations, and termination codon generation in the rdxA gene are common forms of metronidazole resistance [8, 15, 49]. In addition, mutations in other genes such as rpsU and sodB have been observed in metronidazole‐resistant strains, suggesting polygenic synergy in resistance formation [50, 51]. Regarding metabolic pathway regulation, studies have shown that resistant strains reprogram metabolic pathways to reduce drug activation efficiency or enhance resistance through mechanisms such as biofilm formation [43, 52].

Environmental factors can also affect the expression and transmission of metronidazole resistance genes. For example, antibiotic residues in environments such as sewage treatment plants and farmland promote the spread of resistance genes [53, 54]. In animal‐derived strains, such as *H. pylori*–like strains in pig stomachs, frxA gene mutations related to metronidazole resistance have also been detected [55].

Moreover, strains resistant to metronidazole frequently demonstrate physiological alterations, including irregularities in the regulation of iron metabolism and modifications in the redox state. This observation implies that the regulation of metabolic pathways is crucial in the development of resistance [41, 50].

In conclusion, the resistance of *H. pylori* to metronidazole is primarily attributed to mutations in the rdxA and frxA genes, which result in diminished reductase activity. This resistance is further associated with alterations in metabolic pathways and physiological conditions. The advent of genetic testing technologies offers a robust method for predicting resistance, whereas environmental and host‐related factors play a significant role in the dissemination and regulation of resistance gene expression.

### 4.3. Other Antibiotic Resistance Genes and Mechanisms

In addition to clarithromycin and metronidazole, *H. pylori* resistance to amoxicillin and quinolone antibiotics is increasingly severe, with their molecular mechanisms also widely studied. Amoxicillin resistance is mainly associated with pbp1 gene mutations; pbp1 encodes penicillin‐binding protein responsible for cell wall synthesis, and mutations reduce drug binding capacity, producing resistance [15, 44, 56]. Studies have shown that multiple mutation sites in the pbp1 gene are closely associated with amoxicillin resistance, but resistance rates are relatively low and often accompanied by other resistance genes [8, 57].

The primary factor contributing to quinolone resistance is the occurrence of specific mutations within the gyrA gene, responsible for encoding the A subunit of DNA gyrase. Notably, frequent mutation sites involve amino acid alterations at Positions 87 and 91, with examples including N87K, D91N, and D91G, which are associated with a marked increase in resistance levels [15, 37, 58]. Additionally, research has indicated that mutations in the gyrB gene may also contribute synergistically to quinolone resistance [37]. The presence of mutations in both the gyrA and gyrB genes shows a strong correlation with the clinical resistance phenotype, thus making genetic testing a pivotal approach for diagnosing quinolone resistance [15, 37]. Genotyping is now recognized as a robust tool for forecasting quinolone resistance, providing valuable support for the development of personalized clinical treatment plans.

Concerning the mechanisms underlying MDR, *H. pylori* frequently demonstrates phenotypes of MDR that are associated with various mutations in genes and synergistic interactions among these genes [44, 48, 50]. Beyond mutations in target genes, the overexpression of drug efflux pumps has also been implicated in the development of MDR [47]. Genomic analyses have shown that strains exhibiting MDR possess an array of resistance genes alongside mobile genetic elements, which not only augment their resistance abilities but also enhance the potential for gene dissemination [59].

Furthermore, the mechanisms underlying clarithromycin resistance in non–*H. pylori* pathogens, including *Mycobacterium abscessus*, have been investigated. This research has identified mutations in the rrl gene and heightened expression of genes associated with efflux pumps [47, 60, 61]. These findings offer valuable comparative insights into the various resistance mechanisms.

In conclusion, alongside conventional mutations in target genes, genes associated with MDR and mechanisms of drug efflux collaboratively establish a multifaceted resistance network in *H. pylori*. A thorough examination of these mechanisms holds considerable importance for the diagnosis and therapeutic management of resistant strains. Major antibiotics, target genes, and resistance mechanisms in *H. pylori* are detailed in Table 1.

**Table 1:** Major antibiotics, target genes, and resistance mechanisms in *Helicobacter pylori*.

Antibiotic class	Target gene	Key mutations	Resistance mechanism
Clarithromycin	23S rRNA	A2146G, A2147G	Point mutations alter clarithromycin binding sites on 23S rRNA, reducing drug affinity
Metronidazole	rdxA and frxA	Point mutations, frameshift mutations, premature stop codons	RdxA/frxA mutations reduce metronidazole reductase activity, preventing drug activation; metabolic pathway reprogramming
Amoxicillin	pbp1	Multiple site mutations in pbp1 gene	Pbp1 mutations decrease affinity of penicillin‐binding proteins to the drug, impairing cell wall synthesis inhibition
Quinolones (levofloxacin)	gyrA and gyrB	gyrA: N87K, D91N, and D91G (amino acid Positions 87 and 91)	Mutations in DNA gyrase A subunit reduce quinolone binding, impairing DNA replication inhibition
Tetracycline	16S rRNA	Mutations in 16S rRNA gene	Mutations alter tetracycline binding to 30S ribosomal subunit, reducing protein synthesis inhibition
Multi‐drug Resistance	Multiple genes (hefA and efp)	Various mutations in multiple resistance genes	Overexpression of drug efflux pumps; synergistic effects of multiple resistance gene mutations; mobile genetic elements (plasmids, integrons, and transposons)

### 4.4. Diversity and Evolutionary Dynamics of Resistance Genotypes

The genotypes associated with resistance in *H. pylori* demonstrate considerable diversity and intricate evolutionary patterns, with their distribution being notably affected by geographical, host, and environmental factors [8, 48, 62]. On a global scale, *H. pylori* is categorized into various subgroups according to genetic traits, wherein the resistance genotypes display a distinct distribution that is often restricted by geography [62]. Notably, the prevalence of clarithromycin resistance genotypes is markedly higher in regions such as Asia, Europe, and Oceania compared with other parts of the world [62].

With respect to the frequencies of gene mutations, it has been observed that hot spots for mutations related to resistance, such as A2147G in 23S rRNA and the mutations at positions 87 and 91 in gyrA, exhibit varying frequencies across different geographical regions and bacterial strains [15, 38]. This variation is indicative of the distinct local selection pressures that influence these mutations. Furthermore, the association between virulence genotypes, such as cagA and vacA, and the resistance genotypes present in various strains points to the notion that the genetic background of a strain plays a significant role in the evolution of resistance [42, 63].

Horizontal gene transfer is an important mechanism for resistance gene dissemination, especially promoting resistance gene spread through mobile genetic elements such as plasmids, integrons, and transposons [59, 64]. Studies have shown that resistance genes spread rapidly between different strains and environments through horizontal transfer, facilitating the rapid spread of resistance [59]. In addition, combined effects of host factors such as metabolic enzyme genotypes (CYP2C19) and bacterial virulence factor genotypes may also indirectly affect resistance gene selection and strain evolution [63].

In the process of evolving resistance genotypes, bacterial strains obtain compensatory mutations via multiple mechanisms, which serve to mitigate the physiological burdens associated with resistance and improve their adaptability [43, 65]. Concurrently, the pressures imposed by antibiotic exposure and ecological factors in their surroundings propel the ongoing accumulation and diversification of resistance genes [53, 66].

In conclusion, the genotypes associated with resistance in *H. pylori* display considerable variability and dynamic evolutionary traits. The interplay of geographical environments, host‐specific factors, and horizontal gene transfer collectively influences the distribution and spread of resistance genes. Gaining insights into these evolutionary processes is essential for establishing regional resistance surveillance and tailoring treatment approaches that are personalized to individual patients.

## 5. Advances in Research on the Correspondence Between Antibiotic Resistance Genotypes and Phenotypes of *H. pylori*

### 5.1. Current Status of Research on Phenotype–Genotype Consistency

Numerous investigations have demonstrated a significant correlation between antibiotic resistance phenotypes and particular mutations in resistance genes. For instance, in strains of *Staphylococcus epidermidis* associated with endophthalmitis, the genotypic and phenotypic characteristics regarding resistance to methicillin and erythromycin exhibited nearly perfect agreement, as indicated by kappa values of 0.89 and 0.88, respectively [67]. Approximately 98% of the strains that possessed resistance genes for erythromycin and methicillin displayed corresponding resistance phenotypes [67]. In a similar vein, research concerning *H. pylori* resistance revealed a strong correspondence between genotypic identification and phenotypic resistance to clarithromycin and levofloxacin, with kappa values reaching 0.824 and 0.895, respectively [15]. Furthermore, the identification of resistance genes such as CTX‐M, NDM‐1, ermB, and aac (6′)‐Ib in strains responsible for hospital‐acquired infections, including *Escherichia coli* and *Pseudomonas aeruginosa*, was also found to be closely linked to the corresponding resistance phenotypes [68]. These findings underscore the notion that the presence of specific mutations or resistance genes often constitutes the primary genetic foundation for the associated resistance phenotypes, thereby offering a theoretical framework for the judicious application of genotype testing in clinical settings. Genotype to phenotype correspondence and clinical relevance of resistance mutations are detailed in Table 2.

**Table 2:** Genotype to phenotype correspondence and clinical relevance of resistance mutations in *Helicobacter pylori*.

Antibiotic class	Target gene	Genotype–phenotype correspondence	Clinical relevance
Clarithromycin	23S rRNA	•High correlation (The kappa coefficient: 0.703–0.867 [8, 15, 38, 50]) •A2146G/A2147G mutations reliably predict resistance •Strong genotype–phenotype concordance	•Primary biomarker for treatment guidance •Pretreatment molecular testing recommended for first‐line therapy •High clinical utility for personalized treatment
Metronidazole	rdxA and frxA	•Low correlation (The kappa coefficient < 0.200 [8, 69]) •Mutations do not always confer resistance •Complex mechanisms involving metabolic pathway changes •Involves other genes: rpsU, sodB	•Limited clinical predictive value •Phenotypic testing remains the gold standard •High‐dose regimens considered for resistant strains
Amoxicillin	pbp1	•Moderate correlation •High‐level resistance requires multiple concurrent mutations •Low resistance rates (2%–6%) in most regions	•Still effective for most populations •Regional surveillance needed (e.g., Egypt: 95% resistance) •Limited alternative options for penicillin‐allergic patients
Levofloxacin	gyrA and gyrB	•High correlation (The kappa coefficient: 0.664–0.895 [8, 15, 38, 50]) •N87K, D91N, D91G mutations are predictive •Good genotype–phenotype concordance	•Useful for selecting salvage therapy regimens
			•Molecular testing guides second‐line treatment
			•Rising resistance rates require ongoing monitoring
Tetracycline	16S rRNA	•Moderate to high correlation •16S rRNA mutations associated with resistance •Low global resistance rates (< 5%)	•Component of bismuth quadruple therapy
			•Still effective in most regions
			•Limited clinical need for routine testing
Multi‐drug Resistance	Multiple genes (hefA and efp)	•Complex mechanisms involving multiple gene interactions •Overexpression of efflux pumps (hefA) •Mobile genetic elements: plasmids and integrons •Synergistic effects of mutations	•Major clinical challenge •Comprehensive susceptibility testing required •Development of novel therapeutic strategies needed

However, there are also cases of inconsistent phenotypes and genotypes. For example, some *Staphylococcus aureus* strains carry resistance genes but show phenotypic sensitivity or vice versa; this discrepancy may result from silent expression of resistance genes, polygenic regulation, or complex resistance mechanisms [16]. In *H. pylori*, the correlation between metronidazole resistance phenotype and rdxA gene mutations is poor, suggesting that this resistance mechanism may involve other factors [69]. Other studies have pointed out that the expression of environmental resistance genes and phenotypic resistance is affected by microbial community succession and environmental factors, leading to genotype–phenotype differences [70].

Overall, although most studies confirm high correspondence between resistance genotypes and phenotypes, attention should still be paid to factors such as individual gene silencing, polygenic cumulative effects, and environmental regulation on resistance phenotypes, prompting further in‐depth analysis of genetic and phenotypic resistance mechanisms in subsequent research.

### 5.2. Factors Affecting Phenotype–Genotype Correspondence

The main factors affecting phenotype–genotype correspondence include gene expression regulation, environmental and host factors, and limitations of detection technologies.

Firstly, gene expression regulation results in different expression levels of the same resistance gene in different strains, thereby affecting resistance phenotypes. Some resistance genes may be in a silent state, not expressed or expressed at levels insufficient to produce resistance phenotypes [16]. In addition, some resistance may result from polygenic synergy, and the presence of a single gene cannot fully explain the resistance phenotype [67].

Secondly, various environmental influences, including contamination by heavy metals, remnants of antibiotics, and the immune status of the host, significantly impact the manifestation of resistance phenotypes. For instance, the combined stressors of environmental heavy metals and antibiotic exposure can lead to an increase in the prevalence of resistance genes and mobile genetic elements, thereby amplifying the proliferation of resistant bacterial strains [71, 72]. Additionally, host characteristics such as age, sex, and overall health status have been shown to influence the resistance rates of *H. pylori [* *8*, 36]. Notably, elevated resistance rates have been observed among older adults and female patients, indicating that the physiological condition of the host plays a crucial role in exerting selective pressure on resistant microbial populations [8, 36].

Finally, the sensitivity and specificity limitations of detection technologies affect correlation analysis between resistance genes and phenotypes. Traditional culture methods require long time and may miss subdominant resistant strains; molecular detection is rapid but limited by known resistance gene databases and cannot reflect gene expression status [73, 74]. Emerging technologies such as combined phenotypic and genotypic detection and RNA expression analysis have improved resistance detection accuracy [75, 76].

Therefore, the complexity of gene expression regulation, diversity of environment and hosts, and insufficient detection technologies are the main reasons for incomplete consistency between genotypes and phenotypes. Future research should comprehensively consider these factors to improve resistance assessment.

### 5.3. Guiding Significance of Genotype Testing in Clinical Application

Genotyping assays play a crucial role in informing personalized antibiotic therapy within clinical settings. For instance, in *H. pylori* case studies, these tests are capable of swiftly forecasting resistance to clarithromycin and levofloxacin, thereby aiding clinicians in choosing more efficacious eradication protocols and enhancing overall treatment success rates [8, 15]. Research indicates that treatment strategies tailored based on genotypic data can markedly diminish the selective pressure exerted on resistant bacterial strains, consequently lowering the incidence of MDR [11].

Furthermore, the assessment of genotypes significantly enhances both the rates of eradication and the effectiveness of treatment strategies. A case in point is the management of *H. pylori*, where the integration of genotype testing with drug sensitivity profiles enables the formulation of tailored antibiotic therapies, resulting in markedly improved eradication rates [77]. Similar benefits are observed in the context of other bacterial infections, including *Acinetobacter baumannii and Escherichia coli*, where genotype data serve as a critical foundation for modifying antibiotic treatment protocols [78, 79].

In the foreseeable future, the integration of genotypic and phenotypic detection methodologies is poised to emerge as a significant trend in the realm of clinical diagnostics. The amalgamation of genetic mutations, gene expression profiles, and phenotypic resistance assessments can offer a more comprehensive and precise evaluation of resistance status [73, 80]. Nevertheless, this approach to combined testing encounters several obstacles, including the complexities associated with data interpretation, elevated costs, and a lack of adequate technical standardization [74]. Furthermore, with the continuous advancements in molecular diagnostic technologies and the incorporation of artificial intelligence, genotype testing is anticipated to evolve into a more rapid, accurate, and cost‐effective process [81]. This evolution will facilitate real‐time resistance monitoring and enable personalized medication strategies in clinical settings [81].

In conclusion, the implementation of genotype testing plays a crucial role in facilitating personalized antibiotic therapies within clinical settings, leading to enhanced eradication rates and a decrease in the emergence of resistance. Looking ahead, the integration of both genotypic and phenotypic testing methods is anticipated to represent a significant advancement in the management of antibiotic resistance; however, various technical and practical challenges must still be addressed.

## 6. Discussion

Research into the antibiotic resistance of *H. pylori* has progressed into a comprehensive and systematic phase. This evolution has unveiled a complex array of phenotypic and genotypic variations that illuminate the intricate nature of its resistance mechanisms. From a scholarly viewpoint, the present investigations into the relationship between mutations associated with clarithromycin and levofloxacin resistance and observable phenotypic resistance have underscored the significant role of these genetic alterations as indicators of resistance. This insight serves as a foundational scientific basis for clinical diagnostics and therapeutic strategies. Nevertheless, certain discrepancies observed between phenotypic and genotypic expressions indicate that resistance mechanisms cannot be solely attributed to individual gene mutations. Instead, they are likely influenced by a multifaceted interplay of polygenic interactions, regulatory mechanisms of virulence‐associated genes and efflux pump genes expression, metabolic pathway regulation, physiological alterations of the resistant strains, and environmental and host‐related factors [40–43, 46, 47, 50, 52].

In light of this context, the swift advancement of molecular detection technologies has emerged as a pivotal catalyst for enhancing research on resistance and its clinical applications [21, 28, 29]. Genotypic testing not only boosts the effectiveness of identifying resistant strains but also offers targeted recommendations for developing personalized treatment plans. This technological edge mitigates the risks of resistance associated with empirical therapy, facilitating the shift in *H. pylori* eradication strategies from conventional empirical approaches to precision medicine [25, 28]. Consequently, this transition significantly enhances treatment success rates and elevates patients′ quality of life.

Nonetheless, previous studies have predominantly concentrated on the direct relationships between genetic mutations and observable traits, often overlooking the influence of gene expression regulatory networks, alterations in the microenvironment, and the responses of the host immune system on resistance mechanisms. It is essential for forthcoming investigations to incorporate multiomics data to develop multifaceted association models that encompass gene expression, protein modifications, metabolic regulation, and factors related to the host. Such an approach will facilitate a more thorough exploration of resistance mechanisms. Additionally, this strategy will not only address the shortcomings associated with genotype assessments but also unveil novel targets for resistance, thereby fostering the advancement of innovative therapeutic strategies.

At the same time, balancing perspectives from different studies is particularly crucial. On one hand, we should fully affirm the clinical application value of genotype testing and promote it as a standard tool in *H. pylori* infection management; on the other hand, we should be vigilant about potential diagnostic biases from relying solely on genotype data, emphasizing comprehensive judgment combining phenotypic testing and clinical manifestations. Research teams should strengthen data sharing and multicenter collaboration, using large sample, multiregional clinical data to validate research findings and improve the generalizability and clinical translation capability of studies.

## 7. Conclusion

In conclusion, the investigation into antibiotic resistance in *H. pylori* is currently undergoing a transformation, evolving from a focus on the localization of gene mutations to a more integrated analysis that encompasses multiple factors. Moving forward, leveraging cutting‐edge molecular techniques and fostering interdisciplinary collaboration will be essential in constructing more accurate and comprehensive models of resistance mechanisms. Such advancements will lay a robust scientific groundwork for the development of personalized eradication treatment approaches, thereby elevating the prevention and management of *H. pylori* infections to unprecedented levels. This progress will not only be crucial in curtailing the dissemination of resistant strains but will also markedly enhance patient treatment outcomes and prognoses, ultimately embodying the fundamental principles of precision medicine development.

**Nomenclature**
MDR: multidrug resistance
MIC: minimum inhibitory concentration
NGS: next‐generation sequencing
PCR: polymerase chain reaction
WGS: whole genome sequencing

## Author Contributions

We hereby confirm and acknowledge that all authors have contributed significantly to the work presented in this manuscript. Hui Gong was responsible for the drafting of the manuscript. Chang Ping Li contributed to the conception of the review and its critical revision for important intellectual content; Yan Chun Huang, Bo Yang, Zhen Yu Song, Xue Qiong Xu, Fang Cui Xiao and Rong Peng were responsible for the literature analysis and interpretation.

## Funding

This study was supported by the Chengdu Longquanyi District Health Scientific Research Fund in 2024, Project No. WJKY2024003.

## Disclosure

All authors have read and approved the final version of the manuscript and are in full agreement with its content.

## Ethics Statement

The authors have nothing to report.

## Conflicts of Interest

The authors declare no conflicts of interest.

## Data Availability

Data sharing is not applicable to this article as no datasets were generated or analyzed during the current study.

## References

[1] Ahmed N. , Zameer S. , Ghazanfar S. , Islam Z. , Jatoi H. N. , and Khan M. , A short Communication Regarding the Trends and Prevalence of *H. Pylori* in Unselected Gastric Mucosal Biopsy Samples at a Tertiary Care Hospital in Karachi, Pakistan: A Five-Year Retrospective Study, Journal of the Pakistan Medical Association. (2025) 75, no. 1, 100–102, 10.47391/JPMA.10957, 39828837. 39828837

[2] Zvidi I. , Dickman R. , and Boltin D. , Disparity in *Helicobacter pylori* Positivity Among Israeli Adults With Uninvestigated Dyspepsia in an Urban Setting With Mixed Ethnicity, Israel Medical Association Journal. (2023) 25, no. 11, 729–734, 37980617. 37980617

[3] Rkain M. , Bahari H. , Hamami A. , Elouali A. , and Babakhouya A. , Epidemiological, Clinical, and Evolutionary Profile of *Helicobacter pylori* Infection in the Pediatric Population of the Eastern Region of Morocco: A Series of 118 Cases, Cureus. (2025) 17, no. 2, e79449, 10.7759/cureus.79449, 40130108. PMC11931978 40130108

[4] Morais S. , Costa A. , Albuquerque G. , Araújo N. , Tsugane S. , Hidaka A. , Hamada G. S. , Ye W. , Plymoth A. , Leja M. , Gasenko E. , Zaridze D. , Maximovich D. , Malekzadeh R. , Derakhshan M. H. , Pelucchi C. , Negri E. , Camargo M. C. , Curado M. P. , Vioque J. , Zhang Z. F. , la Vecchia C. , Boffetta P. , and Lunet N. , "True" *Helicobacter pylori* Infection and Non-Cardia Gastric Cancer: A Pooled Analysis Within the Stomach Cancer Pooling (StoP) Project, Helicobacter. (2022) 27, no. 3, e12883, 10.1111/hel.12883, 35235224. PMC13523715 35235224

[5] Mamun T. I. , Younus S. , and Rahman M. H. , Gastric Cancer-Epidemiology, Modifiable and Non-Modifiable Risk Factors, Challenges and Opportunities: An Updated Review, Cancer Treatment and Research Communications. (2024) 41, 100845, 10.1016/j.ctarc.2024.100845. 39357127

[6] Choe Y. , Antibiotic Resistance and *Helicobacter pylori* Eradication Therapy, Korean Journal of Helicobacter and Upper Gastrointestinal Research. (2023) 23, no. 3, 218–221, 10.7704/kjhugr.2023.0044, 40502673. PMC11967541 40502673

[7] Botija G. , García Rodríguez C. , Recio Linares A. , Campelo Gutiérrez C. , Pérez-Fernández E. , and Barrio M. A. , Antibiotic Resistances and Eradication Rates in *Helicobacter pylori* Infection, Anales de Pediatría. (2021) 95, no. 6, 431–437, 10.1016/j.anpedi.2020.10.006, 33334694. 34810153

[8] Hu S. , Zhou Y. , Deng Y. , Bo Y. , Chen X. , Yang W. , Shi R. , Zhao W. , Hou Z. , Hu J. , Liu J. , Zhang X. , Yong H. , Wang P. , Li F. , Qi H. , Wang X. , Jin L. , Cui T. , Yong H. , Li X. , Yang B. , Yu Y. , Ma B. , Fu L. , Wang X. , Ma Z. , Tang N. , You Y. , Guo J. , Yu X. , Yao L. , Gao R. , Li Y. , Xin R. , Liu J. , Cao Z. , Li H. , Ma L. , Ma S. , Cao Y. , Tang Y. , Liu J. , Hao Q. , Li X. , Li X. , Mu R. , Niu M. , Su X. , and Gao H. , Characteristics of Phenotypic Antibiotic Resistance of *Helicobacter pylori* and Its Correlation With Genotypic Antibiotic Resistance: A Retrospective Study in Ningxia, Helicobacter. (2023) 28, no. 3, e12960, 10.1111/hel.12960, 37042045. 37042045

[9] Qian X. , Gao B. , Chen Z. , Zhang Z. , and Jiang Z. , Vonoprazan vs. High-Dose Esomeprazole in Bismuth-Containing Quadruple Therapy for *Helicobacter pylori* Rescue Treatment: A Retrospective Cohort Study, Gut Pathogens. (2025) 17, no. 1, 10.1186/s13099-025-00741-0, 40859263. PMC12381992 40859263

[10] Han Z. , Li Y. , Kong Q. , Liu J. , Wang J. , Wan M. , Lin M. , Lin B. , Zhang W. , Ding Y. , Wang S. , Mu Y. , Duan M. , Zuo X. , and Li Y. Q. , Efficacy of Bismuth for Antibiotic-Resistant *Helicobacter pylori* Strains Eradication: A Systematic Review and Meta-Analysis, Helicobacter. (2022) 27, no. 6, e12930, 10.1111/hel.12930, 36156332. 36156332

[11] Gao C. , Du S. Y. , Fang L. , Fan Y. H. , Song A. P. , and Chen H. , Eradication Treatment of *Helicobacter pylori* Infection Based on Molecular Pathologic Antibiotic Resistance, Infection and Drug Resistance. (2020) 13, 69–79, 10.2147/IDR.S232169, 32021321. PMC6954831 32021321

[12] Gong E. J. , Ahn J. Y. , Kim J. M. , Lee S. M. , Na H. K. , Lee J. H. , Jung K. W. , Choi K. D. , Kim D. H. , Song H. J. , Lee G. H. , Kim S. W. , and Jung H. Y. , Genotypic and Phenotypic Resistance to Clarithromycin in *Helicobacter pylori* Strains, Journal of Clinical Medicine. (2020) 9, no. 6, 10.3390/jcm9061930, 32575584. PMC7356929 32575584

[13] Camorlinga-Ponce M. , Gómez-Delgado A. , Aguilar-Zamora E. , Torres R. C. , Giono-Cerezo S. , Escobar-Ogaz A. , and Torres J. , Phenotypic and Genotypic Antibiotic Resistance Patterns in *Helicobacter pylori* Strains From Ethnically Diverse Population in México, Frontiers in Cellular and Infection Microbiology. (2021) 10, 539115, 10.3389/fcimb.2020.539115, 33643927. PMC7905308 33643927

[14] Al-Hyassat M. M. , Al-Daghistani H. I. , Abu-Niaaj L. F. , Zein S. , and Al-Qaisi T. , Allelic Variation of *Helicobacter pylori* vacA Gene and Its Association With Gastric Pathologies in Clinical Samples Collected in Jordan, Microorganisms. (2025) 13, no. 8, 10.3390/microorganisms13081841, 40871345. PMC12388764 40871345

[15] Xiong M. , Mohammed Aljaberi H. S. , Khalid Ansari N. , Sun Y. , Yin S. , Nasifu L. , Sun H. , Xu T. , Pan Y. , Nie Z. , Liu C. , Zhang Z. , Jiang Z. , Wang S. , and He B. , Phenotype and Genotype Analysis for *Helicobacter pylori* Antibiotic Resistance in Outpatients: A Retrospective Study, Microbiology Spectrum. (2023) 11, no. 5, e0055023, 10.1128/spectrum.00550-23, 37732751. PMC10580949 37732751

[16] Rasheed H. , Ijaz M. , Ahmed A. , Javed M. U. , Shah S. F. A. , and Anwaar F. , Discrepancies Between Phenotypic and Genotypic Identification Methods of Antibiotic Resistant Genes Harboring *Staphylococcus aureus* , Microbial Pathogenesis. (2023) 184, 106342, 10.1016/j.micpath.2023.106342, 37704062. 37704062

[17] Chew K. L. , Tan K. X. , Abu Bakar N. A. B. , Lin R. , and Teo J. W. P. , Genotypic Diversity and Antimicrobial Resistance Phenotype of Carbapenem-Resistant and Carbapenem-Susceptible *Acinetobacter* Species Isolates, Pathology. (2025) 57, no. 5, 637–642, 10.1016/j.pathol.2024.12.647, 40204623. 40204623

[18] Bačić A. , Milivojević V. , Petković I. , Kekić D. , Gajić I. , Medić Brkić B. , Popadić D. , Milosavljević T. , and Rajilić-Stojanović M. , In Search for Reasons Behind *Helicobacter pylori* Eradication Failure-Assessment of the Antibiotics Resistance Rate and Co-Existence of *Helicobacter pylori* With *Candida* Species, Journal of Fungi. (2023) 9, no. 3, 10.3390/jof9030328. PMC10056355 36983496

[19] Metwally M. , Ragab R. , Abdel Hamid H. S. , Emara N. , and Elkholy H. , *Helicobacter pylori* Antibiotic Resistance in Egypt: A Single-Center Study, Infection and Drug Resistance. (2022) 15, 5905–5913, 10.2147/IDR.S386082, 36312439. PMC9553238 36312439

[20] Lee J. H. , Kwon Y. H. , Kim S. U. , Park J. H. , Cho J. H. , Bae J. H. , and Jeon S. W. , Antibiotic Resistance of Helicobacter pylori: Current Trends in Daegu-Kyungpook, Korean Journal of Helicobacter and Upper Gastrointestinal Research. (2025) 25, no. 2, 146–151, 10.7704/kjhugr.2025.0010. PMC12173591 40550546

[21] Dumitru E. , Alexandrescu L. , Hanu A. C. , Tocia C. , Cozaru G. C. , Mitroi A. F. , Brînzan C. , A?chie M. , and Dumitru I. M. , Genetic Antibiotic Resistance of *Helicobacter pylori* in South-Eastern Romania, Journal of Gastrointestinal & Liver Diseases. (2020) 29, no. 1, 19–25, 10.15403/jgld-758, 32176750. 32176750

[22] Maev I. V. , Andreev D. N. , Fomenko A. K. , Podporin M. S. , Lyamina S. V. , Zaborovsky A. V. , Khimina I. N. , Cheremushkin S. V. , Bagdasarian A. S. , Cheremushkina N. V. , Lobanova E. G. , Tsarev V. N. , and Zayratyants O. V. , Trends of Antibiotic Resistance of *Helicobacter pylori* in Moscow, Terapevticheskii Arkhiv. (2025) 97, no. 2, 163–168, 10.26442/00403660.2025.02.203193, 40237753. 40237753

[23] Xu H. , Yun J. , Li R. , Ma X. , Gou L. , Che T. , and Zhang D. , Antibiotics Resistance Prevalence of *Helicobacter pylori* Strains in Northwest China, Infection and Drug Resistance. (2022) 15, 5519–5528, 10.2147/IDR.S383444, 36172620. PMC9512286 36172620

[24] Zanotti G. and Cendron L. , Structural Aspects of *Helicobacter pylori* Antibiotic Resistance, Advances in Microbiology, Infectious Diseases and Public Health. (2019) 1149, 227–241, 10.1007/5584_2019_368, 31016632. 31016632

[25] Bujanda L. , Nyssen O. P. , Ramos J. , Bordin D. S. , Tepes B. , Perez-Aisa A. , Pavoni M. , Castro-Fernandez M. , Lerang F. , Leja M. , Rodrigo L. , Rokkas T. , Kupcinskas J. , Jonaitis L. , Shvets O. , Gasbarrini A. , Simsek H. , Phull P. S. , Buzás G. M. , Machado J. C. , Boltin D. , Boyanova L. , Tonkić A. , Marlicz W. , Venerito M. , Vologzanina L. , Fadieienko G. D. , Fiorini G. , Resina E. , Muñoz R. , Cano-Català A. , Puig I. , García-Morales N. , Hernández L. , Moreira L. , Megraud F. , Morain C. O. , Montes M. , and Gisbert J. P. , Effectiveness of *Helicobacter pylori* Treatments According to Antibiotic Resistance, Official Journal of the American College of Gastroenterology. (2024) 119, no. 4, 646–654, 10.14309/ajg.0000000000002600. 37983769

[26] Vu T. B. , Tran T. N. Q. , Tran T. Q. A. , Vu D. L. , and Hoang V. T. , Antibiotic Resistance of Helicobacter pylori in Patients With Peptic Ulcer, Medicina. (2023) 59, no. 1, 10.3390/medicina59010006. PMC9864273 36676631

[27] Fauzia K. A. and Tuan V. P. , Rising Resistance: Antibiotic Choices for *Helicobacter pylori* Infection, Lancet Gastroenterology & Hepatology. (2024) 9, no. 1, 7–8, 10.1016/S2468-1253(23)00354-0, 37972626. 37972626

[28] Brennan D. , O′Morain C. , McNamara D. , and Smith S. M. , Molecular Detection of Antibiotic-Resistant *Helicobacter pylori* , Methods in Molecular Biology. (2021) 2283, 29–36, 10.1007/978-1-0716-1302-3_4, 33765306. 33765306

[29] Lv Y. T. , Li D. , Zhang D. Y. , Chen S. J. , Chen R. X. , Wang Y. , Yang W. Z. , Gao L. , Zeng J. T. , Xiong J. X. , Huang Q. Y. , Huang J. , Zhang Q. G. , Chen J. J. , and Bai F. H. , *Helicobacter pylori* Resistance in Hainan Province, China: Investigating Phenotypes and Genotypes Through Whole-Genome Sequencing, Frontiers in Cellular and Infection Microbiology. (2024) 14, 1505166, 10.3389/fcimb.2024.1505166, 39742338. PMC11685075 39742338

[30] Ng H. Y. , Leung W. K. , and Cheung K. S. , Antibiotic Resistance, Susceptibility Testing and Stewardship in *Helicobacter pylori* Infection, International Journal of Molecular Sciences. (2023) 24, no. 14, 11708, 10.3390/ijms241411708, 37511471. PMC10380565 37511471

[31] Mustafa A. S. , Whole Genome Sequencing: Applications in Clinical Bacteriology, Medical Principles and Practice. (2024) 33, no. 3, 185–197, 10.1159/000538002, 38402870. PMC11221363 38402870

[32] Huang Y. H. and Jiang S. , Combining Phenotypic and Genotypic Methods for the Rapid Detection of Antibiotic-Resistant Bacteria in Wastewater Using a Microfluidic Centrifugal Disc, ACS Es&t Engineering. (2025) 5, no. 8, 1943–1952, 10.1021/acsestengg.5c00107, 40809007. PMC12340755 40809007

[33] Bennett I. , Pyne A. L. B. , and McKendry R. A. , Cantilever Sensors for Rapid Optical Antimicrobial Sensitivity Testing, ACS Sensors. (2020) 5, no. 10, 3133–3139, 10.1021/acssensors.0c01216, 32900182. PMC7589985 32900182

[34] Rosłon I. , Japaridze A. , Rodenhuis S. , Hamoen L. , Ghatkesar M. K. , Steeneken P. , Dekker C. , and Alijani F. , Microwell-Enhanced Optical Rapid Antibiotic Susceptibility Testing of Single Bacteria, iScience. (2023) 26, no. 11, 108268, 10.1016/j.isci.2023.108268, 38026160. PMC10654606 38026160

[35] Medakina I. , Tsapkova L. , Polyakova V. , Nikolaev S. , Yanova T. , Dekhnich N. , Khatkov I. , Bordin D. , and Bodunova N. , *Helicobacter pylori* Antibiotic Resistance: Molecular Basis and Diagnostic Methods, International Journal of Molecular Sciences. (2023) 24, no. 11, 10.3390/ijms24119433, 37298385. PMC10253745 37298385

[36] White B. , Winte M. , DeSipio J. , and Phadtare S. , Clinical Factors Implicated in Antibiotic Resistance in *Helicobacter pylori* Patients, Microorganisms. (2022) 10, no. 2, 10.3390/microorganisms10020322, 35208776. PMC8876575 35208776

[37] Ziver-Sarp T. , Yuksel-Mayda P. , Saribas S. , Demiryas S. , Gareayaghi N. , Ergin S. , Tasci I. , Ozbey D. , Bal K. , Erzin Y. , Akkus S. , Bahar-Tokman H. , Demirci M. , Tufan-Kocak B. , and Kocazeybek B. , Point Mutations at gyrA and gyrB Genes of Levofloxacin Resistant *Helicobacter pylori* Strains and Dual Resistance With Clarithromycin, Clinical Laboratory. (2021) 67, no. 10/2021, 10.7754/Clin.Lab.2021.210843, 34655183. 34655183

[38] Cui R. , Song Z. , Suo B. , Tian X. , Xue Y. , Meng L. , Niu Z. , Jin Z. , Zhang H. , and Zhou L. , Correlation Analysis Among Genotype Resistance, Phenotype Resistance and Eradication Effect of *Helicobacter pylori* , Infection and Drug Resistance. (2021) 14, 1747–1756, 10.2147/IDR.S305996, 34012273. PMC8127322 34012273

[39] Marques A. T. , Vítor J. M. B. , Santos A. , Oleastro M. , and Vale F. F. , Trends in *Helicobacter pylori* Resistance to Clarithromycin: From Phenotypic to Genomic Approaches, Microbial Genomics. (2020) 6, no. 3, e000344, 10.1099/mgen.0.000344. PMC7200067 32118532

[40] Alavifard H. , Mirzaei N. , Yadegar A. , Baghaei K. , Smith S. M. , Sadeghi A. , and Zali M. R. , Investigation of Clarithromycin Resistance-Associated Mutations and Virulence Genotypes of *Helicobacter pylori* Isolated From Iranian Population: A Cross-Sectional Study, Current Microbiology. (2021) 78, no. 1, 244–254, 10.1007/s00284-020-02295-7, 33251569. 33251569

[41] Li X. H. , Huang Y. Y. , Lu L. M. , Zhao L. J. , Luo X. K. , Li R. J. , Dai Y. Y. , Qin C. , Huang Y. Q. , and Chen H. , Early Genetic Diagnosis of Clarithromycin Resistance in *Helicobacter pylori* , World Journal of Gastroenterology. (2021) 27, no. 24, 3595–3608, 10.3748/wjg.v27.i24.3595, 34239272. PMC8240046 34239272

[42] Zhao P. , Zhao J. , Shi H. , Meng F. , Yang N. , Dong L. , and Gong J. , Relationship Between Antibiotic Resistance and the cagA and vacA Genotypes Among *Helicobacter pylori* Strain Isolates From Patients in Xi′an, Brazilian Journal of Microbiology. (2023) 54, no. 4, 2773–2780, 10.1007/s42770-023-01133-9, 37779175. PMC10689652 37779175

[43] Rosli N. A. , Al-Maleki A. R. , Loke M. F. , Chua E. G. , Alhoot M. A. , and Vadivelu J. , Polymorphism of Virulence Genes and Biofilm Associated With In Vitro Induced Resistance to Clarithromycin in *Helicobacter pylori* , Gut Pathogens. (2023) 15, no. 1, 10.1186/s13099-023-00579-4, 37898785. PMC10613384 37898785

[44] Hu L. , Zeng X. , Ai Q. , Liu C. , Zhang X. , Chen Y. , Liu L. , Li G. Q. , Alarcón T. , and Long X. , Long-Read- and Short-Read-Based Whole-Genome Sequencing Reveals the Antibiotic Resistance Pattern of *Helicobacter pylori* , Microbiology Spectrum. (2023) 11, no. 3, e0452222, 10.1128/spectrum.04522-22, 37067452. PMC10269496 37067452

[45] Si X. B. , Bi D. Y. , Lan Y. , Zhang S. , and Huo L. Y. , Gastric Juice-Based Genotypic Methods for Diagnosis of *Helicobacter pylori* Infection and Antibiotic Resistance Testing: A Systematic Review and Meta-Analysis, Turkish Journal of Gastroenterology. (2021) 32, no. 1, 53–65, 10.5152/tjg.2020.20025, 33893766. PMC8975514 33893766

[46] Elbaiomy R. G. , Luo X. , Guo R. , Deng S. , du M. , el-Sappah A. H. , Bakeer M. , Azzam M. M. , Elolimy A. A. , Madkour M. , Li Z. , and Zhang Z. , Antibiotic Resistance in *Helicobacter pylori*: A Genetic and Physiological Perspective, Gut Pathogens. (2025) 17, no. 1, 10.1186/s13099-025-00704-5, 40410811. PMC12102891 40410811

[47] Vianna J. S. , Machado D. , Ramis I. B. , Silva F. P. , Bierhals D. V. , Abril M. A. , von Groll A. , Ramos D. F. , Lourenço M. C. S. , Viveiros M. , and da Silva P. E. A. , The Contribution of Efflux Pumps in *Mycobacterium abscessus* Complex Resistance to Clarithromycin, Antibiotics. (2019) 8, no. 3, 10.3390/antibiotics8030153. PMC6784190 31540480

[48] Zhong Z. , Zhang Z. , Wang J. , Hu Y. , Mi Y. , He B. , Zhang Y. , Zhang X. , Xia X. , Huang H. , Lai Y. , Lin M. , Su C. , Zhang Z. , Wu Z. , Lu L. , Zhang B. , Huang S. , Zhong C. , Zeng X. , Peng Y. , Chen G. , Zhang H. , Zhou G. , Liu S. , Yang C. , Yan L. , Chen A. , Zhang G. , Xu P. , Wang S. , Zheng P. , Xu S. , and Gao H. , A Retrospective Study of the Antibiotic-Resistant Phenotypes and Genotypes of *Helicobacter pylori* Strains in China, American Journal of Cancer Research. (2021) 11, no. 10, 5027–5037, 34765309. PMC8569369 34765309

[49] Farzi N. , Yadegar A. , Sadeghi A. , Asadzadeh Aghdaei H. , Marian Smith S. , Raymond J. , Suzuki H. , and Zali M. R. , High Prevalence of Antibiotic Resistance in Iranian *Helicobacter pylori* Isolates: Importance of Functional and Mutational Analysis of Resistance Genes and Virulence Genotyping, Journal of Clinical Medicine. (2019) 8, no. 11, 10.3390/jcm8112004, 31744181. PMC6912791 31744181

[50] Zhou Y. , Zhong Z. , Hu S. , Wang J. , Deng Y. , Li X. , Chen X. , Li X. , Tang Y. , Li X. , Hao Q. , Liu J. , Sang T. , Bo Y. , and Bai F. , A Survey of *Helicobacter pylori* Antibiotic-Resistant Genotypes and Strain Lineages by Whole-Genome Sequencing in China, Antimicrobial Agents and Chemotherapy. (2022) 66, no. 6, e0218821, 10.1128/aac.02188-21, 35652644. PMC9211431 35652644

[51] Li Y. , Huang Z. , Shang Y. , Xie X. , Yang R. , Chen H. , Wang Z. , Xue L. , Pang R. , Zhang J. , Ding Y. , Chen M. , Wang J. , Chen J. , and Wu Q. , Exploration of the Molecular Mechanisms Underlying the Antibiotic Resistance of *Helicobacter pylori*: A Whole-Genome Sequencing-Based Study in Southern China, Helicobacter. (2022) 27, no. 2, e12879, 10.1111/hel.12879, 35124867. 35124867

[52] Gong M. , Han Y. , Wang X. , Tao H. , Meng F. , Hou B. , Sun B. B. , and Wang G. , Effect of Temperature on Metronidazole Resistance in *Helicobacter pylori* , Frontiers in Microbiology. (2021) 12, 681911, 10.3389/fmicb.2021.681911, 34093508. PMC8170400 34093508

[53] Zhang R. , Yang S. , An Y. , Wang Y. , Lei Y. , and Song L. , Antibiotics and Antibiotic Resistance Genes in Landfills: A Review, Science of the Total Environment. (2022) 806, Pt 2, 150647, 10.1016/j.scitotenv.2021.150647, 34597560. 34597560

[54] Weng Z. F. , He Y. Q. , Li G. X. , Wu X. T. , Dai Y. , and Bao P. , Investigation of Antibiotic-Resistant Bacterial Communities and Antibiotic-Resistant Genes in Wastewater Treatment Plants: Removal of Antibiotic-Resistant Genes by the BBR Process, Bulletin of Environmental Contamination and Toxicology. (2022) 108, no. 2, 284–291, 10.1007/s00128-021-03363-9, 34532751. 34532751

[55] Cortez Nunes F. , Taillieu E. , Letra Mateus T. , Teixeira S. , Haesebrouck F. , and Amorim I. , Molecular Detection of Metronidazole and Tetracycline Resistance Genes in *Helicobacter pylori*-Like Positive Gastric Samples From Pigs, Antibiotics. (2023) 12, no. 5, 10.3390/antibiotics12050906. PMC10215371 37237809

[56] Silva K. P. T. and Khare A. , Antibiotic Resistance Mediated by Gene Amplifications, NPJ Antimicrobials and Resistance. (2024) 2, no. 1, 10.1038/s44259-024-00052-5, 39843582. PMC11721125 39843582

[57] Losurdo G. , Mezzapesa M. , Ditonno I. , Piazzolla M. , Pricci M. , Girardi B. , Celiberto F. , Galeano G. , Riezzo G. , Russo F. , Iannone A. , Ierardi E. , and di Leo A. , *Helicobacter pylori* Secondary Antibiotic Resistance After One or More Eradication Failure: A Genotypic Stool Analysis Study, Antibiotics. (2024) 13, no. 4, 10.3390/antibiotics13040336. PMC11047300 38667013

[58] Spiteri J. A. , Zahra G. , Schembri J. , Pisani A. , Borg E. , Spiteri N. , Bianco E. Z. , Caruana P. , Gauci J. , Muscat M. , Barbara C. , and Ellul P. , Identification of Antibiotic Resistance Patterns in *Helicobacter pylori* Strains Isolated From Gastric Biopsies Using Real-Time PCR and Genotypic Analysis, Annals of Gastroenterology. (2021) 34, no. 4, 501–509, 10.20524/aog.2021.0624, 34276188. PMC8276370 34276188

[59] Wang X. , Zhang H. , Yu S. , Li D. , Gillings M. R. , Ren H. , Mao D. , Guo J. , and Luo Y. , Inter-Plasmid Transfer of Antibiotic Resistance Genes Accelerates Antibiotic Resistance in Bacterial Pathogens, ISME Journal. (2024) 18, no. 1, wrad032, 10.1093/ismejo/wrad032, 38366209. PMC10881300 38366209

[60] Guo Q. , Chen J. , Zhang S. , Zou Y. , Zhang Y. , Huang D. , Zhang Z. , Li B. , and Chu H. , Efflux Pumps Contribute to Intrinsic Clarithromycin Resistance in Clinical, *Mycobacterium abscessus* Isolates, Infection and Drug Resistance. (2020) 13, 447–454, 10.2147/IDR.S239850, 32104016. PMC7024787 32104016

[61] Realegeno S. , Mirasol R. , Garner O. B. , and Yang S. , Clinical Whole Genome Sequencing for Clarithromycin and Amikacin Resistance Prediction and Subspecies Identification of *Mycobacterium abscessus* , Journal of Molecular Diagnostics. (2021) 23, no. 11, 1460–1467, 10.1016/j.jmoldx.2021.07.023, 34454109. 34454109

[62] Zhu M. , Xu X. , Cai P. , Wang T. , Zhu M. , Yan C. , Pan Q. , Chen C. , Wu Y. , Zhang G. , and Jin G. , Global Population Structure, Virulence Factors and Antibiotic Resistance of *Helicobacter pylori*: A Pooled Analysis of 4067 Isolates From 76 Countries, Helicobacter. (2025) 30, no. 2, e70025, 10.1111/hel.70025, 40059062. 40059062

[63] Auttajaroon J. , Chotivitayatarakorn P. , Yamaoka Y. , and Vilaichone R. K. , CYP2C19 Genotype, CagA Genotype and Antibiotic Resistant Strain of *Helicobacter pylori* Infection, Asian Pacific Journal of Cancer Prevention. (2019) 20, no. 4, 1243–1247, 10.31557/APJCP.2019.20.4.1243, 31030500. PMC6948911 31030500

[64] Ito Y. , Hashimoto Y. , Suzuki M. , Kaneko N. , Yoshida M. , Nakayama H. , and Tomita H. , The Emergence of Metronidazole-Resistant *Prevotella bivia* Harboring nimK Gene in Japan, Microbiology Spectrum. (2024) 12, no. 10, e0056224, 10.1128/spectrum.00562-24, 39162532. PMC11448248 39162532

[65] Mohiuddin S. G. , Kavousi P. , Figueroa D. , Ghosh S. , and Orman M. A. , The Diverse Phenotypic and Mutational Landscape Induced by Fluoroquinolone Treatment, mSystems. (2025) 10, no. 8, e0071325, 10.1128/msystems.00713-25, 40742148. PMC12363166 40742148

[66] Michaelis C. and Grohmann E. , Horizontal Gene Transfer of Antibiotic Resistance Genes in Biofilms, Antibiotics. (2023) 12, no. 2, 10.3390/antibiotics12020328. PMC9952180 36830238

[67] Laura D. M. , Scott N. L. , Vanner E. A. , Miller D. , and Flynn H. W. , Genotypic and Phenotypic Antibiotic Resistance in *Staphylococcus epidermidis* Endophthalmitis, Ophthalmic Surgery, Lasers and Imaging Retina. (2020) 51, no. 5, S13–S16, 10.3928/23258160-20200108-02, 32484896. 32484896

[68] Sarjana Safain K. , Bhuyan G. S. , Hassan Hasib S. , Islam M. S. , Mahmud-Un-Nabi M. A. , Sultana R. , Tasnim S. , Noor F. A. , Sarker S. K. , Islam M. T. , Rahat A. , Leung D. T. , Domman D. , Manzoor F. , Anwar S. , Majid Bhuiyan M. A. , Chowdhury E. K. , Qadri S. S. , Qadri F. , and Mannoor K. , Genotypic and Phenotypic Profiles of Antibiotic-Resistant Bacteria Isolated From Hospitalised Patients in Bangladesh, Tropical Medicine & International Health. (2021) 26, no. 7, 720–729, 10.1111/tmi.13584, 33838068. 33838068

[69] Wang Y. H. , Wang F. F. , Gong X. L. , Yan L. L. , Zhao Q. Y. , Song Y. P. , Zhao R. L. , He Y. J. , Zhou L. , Liu D. S. , and Xie Y. , Genotype Profiles of *Helicobacter pylori* From Gastric Biopsies and Strains With Antimicrobial-Induced Resistance, Therapeutic Advances in Gastroenterology. (2020) 13, 1756284820952596, 10.1177/1756284820952596, 33029198. PMC7522827 33029198

[70] Sun S. , Geng J. , Ma L. , Sun X. , Qi H. , Wu Y. , and Zhang R. , Changes in Antibiotic Resistance Genotypes and Phenotypes After Two Typical Sewage Disposal Processes, Chemosphere. (2022) 291, Pt 2, 132833, 10.1016/j.chemosphere.2021.132833, 34762888. 34762888

[71] Fu C. X. , Chen C. , Xiang Q. , Wang Y. F. , Wang L. , Qi F. Y. , Zhu D. , Li H. Z. , Cui L. , Hong W. L. , Rillig M. C. , Zhu Y. G. , and Qiao M. , Antibiotic Resistance at Environmental Multi-Media Interfaces Through Integrated Genotype and Phenotype Analysis, Journal of Hazardous Materials. (2024) 480, 136160, 10.1016/j.jhazmat.2024.136160, 39413517. 39413517

[72] Engin A. B. , Engin E. D. , and Engin A. , Effects of Co-Selection of Antibiotic-Resistance and Metal-Resistance Genes on Antibiotic-Resistance Potency of Environmental Bacteria and Related Ecological Risk Factors, Environmental Toxicology and Pharmacology. (2023) 98, 104081, 10.1016/j.etap.2023.104081, 36805463. 36805463

[73] Sun H. , Levenfors J. J. , Brandt C. , and Schnürer A. , Assessing Phenotypic and Genotypic Antibiotic Resistance in *Bacillus*-Related Bacteria Isolated From Biogas Digestates, Ecotoxicology and Environmental Safety. (2025) 291, 117859, 10.1016/j.ecoenv.2025.117859, 39947064. 39947064

[74] Freitas A. R. and Werner G. , Antibiotic Susceptibility Testing for Therapy and Antimicrobial Resistance Surveillance: Genotype Beats Phenotype?, Future Microbiology. (2022) 17, no. 14, 1093–1097, 10.2217/fmb-2022-0109, 35833803. 35833803

[75] Bhattacharyya R. P. , Bandyopadhyay N. , Ma P. , Son S. S. , Liu J. , He L. L. , Wu L. , Khafizov R. , Boykin R. , Cerqueira G. C. , Pironti A. , Rudy R. F. , Patel M. M. , Yang R. , Skerry J. , Nazarian E. , Musser K. A. , Taylor J. , Pierce V. M. , Earl A. M. , Cosimi L. A. , Shoresh N. , Beechem J. , Livny J. , and Hung D. T. , Simultaneous Detection of Genotype and Phenotype Enables Rapid and Accurate Antibiotic Susceptibility Determination, Nature Medicine. (2019) 25, no. 12, 1858–1864, 10.1038/s41591-019-0650-9, 31768064. PMC6930013 31768064

[76] Bonyadian M. , Barati S. , and Mahzounieh M. R. , Phenotypic and Genotypic Characterization of Antibiotic-Resistant in *Escherichia coli* Isolates From Patients With Diarrhea, Iranian Journal of Microbiology. (2019) 11, no. 3, 220–224, 10.18502/ijm.v11i3.1323, 31523405. PMC6711866 31523405

[77] Nakajima S. , Inoue H. , Satake H. , Chatani R. , Ohara M. , Tsubakimoto Y. , Fujii M. , Hasegawa H. , Takahashi K. , Hayafuji K. , and Fujiyama Y. , Two-Dimension Tailor-Made Therapy: A New Salvage Therapy After Multiple Eradication Failures for *Helicobacter pylori* Infection, Gastro Hep Advances. (2022) 1, no. 2, 210–222, 10.1016/j.gastha.2021.11.006, 39131120. PMC11308230 39131120

[78] Wiradiputra M. R. D. , Thirapanmethee K. , Khuntayaporn P. , Wanapaisan P. , and Chomnawang M. T. , Comparative Genotypic Characterization Related to Antibiotic Resistance Phenotypes of Clinical Carbapenem-Resistant *Acinetobacter baumannii* MTC1106 (ST2) and MTC0619 (ST25), BMC Genomics. (2023) 24, no. 1, 10.1186/s12864-023-09734-2, 37978344. PMC10655397 37978344

[79] Ford C. T. , Zenarosa G. L. , Smith K. B. , Brown D. C. , Williams J. , and Janies D. , Genetic Capitalism and Stabilizing Selection of Antimicrobial Resistance Genotypes in *Escherichia coli* , Cladistics. (2020) 36, no. 4, 348–357, 10.1111/cla.12421, 34618971. 34618971

[80] Yang B. , Xin X. , Cao X. , Nasifu L. , Nie Z. , and He B. , Phenotypic and Genotypic Perspectives on Detection Methods for Bacterial Antimicrobial Resistance in a One Health Context: Research Progress and Prospects, Archives of Microbiology. (2024) 206, no. 10, 10.1007/s00203-024-04131-z. 39302440

[81] Theodorakis N. , Feretzakis G. , Hitas C. , Kreouzi M. , Kalantzi S. , Spyridaki A. , Boufeas I. Z. , Sakagianni A. , Paxinou E. , Verykios V. S. , and Nikolaou M. , Antibiotic Resistance in the Elderly: Mechanisms, Risk Factors, and Solutions, Microorganisms. (2024) 12, no. 10, 10.3390/microorganisms12101978, 39458286. PMC11509523 39458286

